# Altered marginal zone and innate-like B cells in aged senescence-accelerated SAMP8 mice with defective IgG1 responses

**DOI:** 10.1038/cddis.2017.351

**Published:** 2017-08-17

**Authors:** Isabel Cortegano, Mercedes Rodríguez, Isabel Martín, Maria Carmen Prado, Carolina Ruíz, Rafael Hortigüela, Mario Alía, Marçal Vilar, Helena Mira, Eva Cano, Mercedes Domínguez, Belén de Andrés, María Luisa Gaspar

**Affiliations:** 1 Centro Nacional de Microbiología, Instituto de Salud Carlos III (ISCIII), Majadahonda, Madrid, Spain; 2 Unidad Funcional de Investigación en Enfermedades Crónicas, Instituto de Salud Carlos III (ISCIII), Majadahonda, 28220 Madrid, Spain; 3 Instituto de Biomedicina de Valencia, Valencia 46010, Spain

## Abstract

Aging has a strong impact on the activity of the immune system, enhancing susceptibility to pathogens and provoking a predominant pre-inflammatory status, whereas dampening responses to vaccines in humans and mice. Here, we demonstrate a loss of marginal zone B lymphocytes (MZ, CD19^+^CD45R^+^CD21^++^CD23^lo^) and a decrease of naive B cells (CD19^+^IgD^+^), whereas there is an enhancement of a CD19^+^CD45R^lo^ innate-like B cell population (B1REL) and the so-called aged B cell compartment (ABC, CD45R^+^CD21^lo^CD23^lo^CD5^−^CD11b^−^) in aged senescence-accelerated (SAMP8) mice but not in aged senescence-resistant (SAMR1) mice. These changes in aged SAMP8 mice were associated with lower IgG isotype levels, displaying low variable gene usage repertoires of the immunoglobulin heavy chain (V_H_) diversity, with a diminution on IgG1-memory B cells (CD11b^−^Gr1^−^CD138^−^IgM^−^IgD^−^CD19^+^CD38^+^IgG1^+^), an increase in T follicular helper (T_FH_, CD4^+^CXCR5^+^PD1^+^) cell numbers, and an altered MOMA-1 (metallophilic macrophages) band in primary follicles. LPS-mediated IgG1 responses were impaired in the B1REL and ABC cell compartments, both *in vitro* and *in vivo*. These data demonstrate the prominent changes to different B cell populations and in structural follicle organization that occur upon aging in SAMP8 mice. These novel results raise new questions regarding the importance of the cellular distribution in the B cell layers, and their effector functions needed to mount a coordinated and effective humoral response.

Age-associated alterations of the immune system include the establishment of a pre-inflammatory state that induces enhanced susceptibility to pathogens and weaker responses to vaccines.^1, 2, 3, 4, 5, 6, 7^ In fact, aging has a strong impact on B cell-dependent responses. Humoral responses from elderly individuals are markedly different from those of adults.^8, 9, 10^ Different studies have demonstrated a loss of B cell progenitors in the bone marrow (BM),^11, 12^ opening interesting questions regarding the possible intrinsic genetic defects carried by B cell precursors and the regulation of the transcription factors that direct B lymphopoiesis.^13, 14, 15^

The composition of splenic B cell compartments differs in aged individuals from those in adult, possibly reflecting the ontogeny of the B cells and the loss of B cell progenitors in the BM on elder individuals.^16^ Moreover, the so-called age-associated B cells (ABCs) appear in the spleen and increase progressively in mice up to 24-month-old.^17, 18^ The ABCs secrete TNF-*α* and mediate the down regulation of B lymphopoiesis in elderly mice,^19^ indicating that this population inhibits the production of B cells and the balance of mature B cell compartments. However, the numbers of follicular B cells (FO) are roughly maintained with age,^20, 21^ apparently due to a slower turnover. Similarly, the innate-like CD19^+^CD45R^lo^ (B1REL) B cells identified by our group, which are related to the B1 cells and their splenic progenitors^22, 23^ (fetal origin, pre-activation state and spontaneous IgM secretion), spontaneously secrete IgG1 and IgA and maintain their number in adult mice for 12 months.^24, 25^ In addition, B1REL cell subset shares phenotypic traits (CD21^lo^CD23^lo^CD5^−^CD11b^−^) with the aforementioned ABC population.

Continuous sister-brother breeding of AKR/J mice led to the generation of several strains prone (SAMP) or resistant (SAMR) to develop an accelerated senescence.^26^ Among them, SAMP8 mice have been widely used as a model for geriatric and neurological disorders,^27, 28, 29^ and display several immune alterations: deficient CD4^+^ T-cell function, low IgG1 in sera, presence of auto-antibodies and impaired responses to viral infection and to granulocyte macrophage colony-stimulating factor (GM-CSF).^2, 7, 30, 31, 32^

Here, we have used the SAMP8 model to analyze the composition and function of the B cell compartments in aged mice (10-month-old), compared with the control strain SAMR1. As expected, an increase in the ABC population was detected. Surprisingly, a substantial loss of marginal zone B cells (MZ) and a striking accumulation of B1REL cells were also found in SAMP8 but not SAMR1 mice, accompanied by an altered follicular organization, with a thicker metallophilic-macrophage band (MOMA-1 band). The accumulated ABCs and B1REL cells from SAMP8 mice, compared with SAMR1 mice, displayed higher proliferation rates *in vivo* with similar apoptosis rates. By contrast, MZ cells from 3-month-old SAMP8 mice had much higher apoptosis than that found on cells from SAMR1 mice. Also, the IgG1-specific humoral response of SAMP8 mice was strongly reduced, coupled to impaired functional maturation of B1REL and B2 cells. Analysis of the V_H_ repertoire used in IgH transcripts from aged SAMP8 mice showed a restricted V_H_-IgG1 repertoire. A profound impairment of terminal differentiation, both at the level of IgG1-memory B cells (memBC) and IgG1-antibody secreting cells (IgG1-ASC), was remarkable in SAMP8 mice. Finally, there was a marked inability of B1REL cells from aged SAMP8 mice to produce *in vitro* and *in vivo* IgG1 in response to LPS, which did not occur in aged-matched SAMR1 mice, whereas antigen-specific T-dependent responses were maintained.

## Results

### Altered distributions of splenic B-cell subsets in aged SAMP8 mice

**Figure 1:**
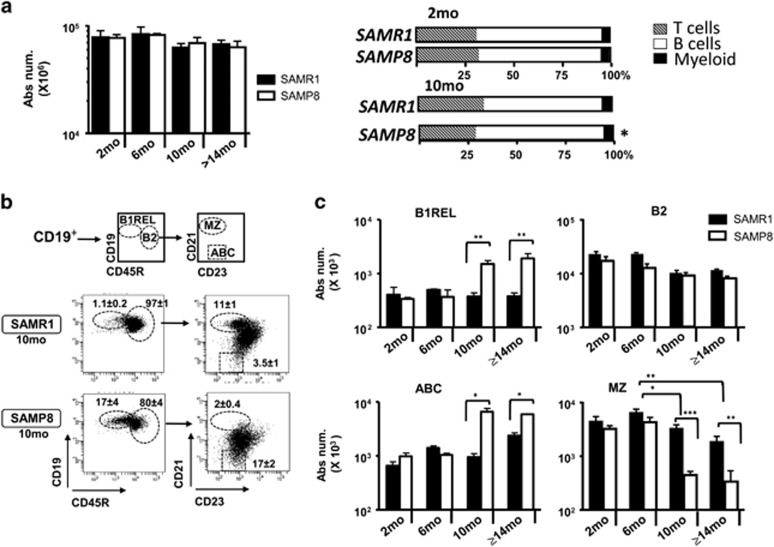
Altered distribution of splenic MZ, B1REL, B2 and ABC cells in aged SAMP8 mice. Splenic cell suspensions from SAMR1 and SAMP8 mice were prepared, counted and stained with the mAbs indicated below for flow cytometry analyses (see ‘Materials and Methods’). (**a**) Left graph, total number of cells recovered from SAMP8 and SAMR1 mice at the ages indicated. Viable cells were calculated on the basis of trypan blue exclusion. Right bar graphs show the frequencies of T, B and myeloid cell subsets from 2-month (mo)-old and 10-month-old SAMP8 and SAMR1 mice, determined by flow cytometry using anti-CD4-PE and anti-CD8-APC (T cells), anti-CD19-PE-Cy7 (B cells) and anti-CD11b-APC-Cy7(myeloid cells). Data are mean±S.E.M. (*n*=4). Comparisons were performed by using a two-tailed Student *t*-test; *P<0.05. (**b**) Splenic cells were stained using anti-CD19-Violet-421, anti-CD45R-PE, anti-CD21-APC and anti-CD23-PE-Cy7. Left dot-plots, CD19^+^B cells were electronically gated, and the B1REL and conventional B2 cell compartments were identified as CD19^+^CD45R^lo^ (B1REL, ellipse) and CD19^+^CD45R^+^ (B2, circle). Right dot-plots, the MZ and ABC cell compartments were identified among gates B2 cells as CD21^++^CD23^lo^ (MZ, ellipse) and CD21^lo^CD23^lo^ (ABC, square). A representative staining of 10-month-old SAMR1 and SAMP8 mice is shown. The numbers inside the plots are the frequencies of each population (mean±S.E.M.; *n*=6). Fluorescence scales are logarithmic. (**c**) The bar graphs show the absolute number/spleen of B1REL, B2, ABC and MZ cells at the indicated ages. These numbers were calculated from the frequencies of each population as described in **b**. Group comparisons were made using a two-tailed Student *t*-test: *P<0.05; **P<0.01; ***P<0.001

We traced the major changes in leukocytes within different hematopoietic organs of SAMP8 and SAMR1 mice. The cellularity and the proportion of myeloid cells in splenic samples were maintained in aged mice of both strains, whereas there was an increase in the B cell compartment and a reduction in the T-cell compartment in samples from aged SAMP8 mice (Figure 1a). There were no differences between aged SAMP8 and SAMR1 mice in terms of the number of B cells and their progenitors in the bone marrow, lymph nodes and peritoneal B-cell subsets (Supplementary Figure S1). Therefore, we focused on the B-cell subsets residing in the spleen. We first traced the innate-like B1REL cells and conventional B2 (CD19^+^CD45R^+^) cells, defined on the basis of CD19/CD45R markers (Figure 1b). These populations were detected at similar frequencies at 2- and 6-month-old, yet by 10-month-old there was an increase in B1REL cells in aged SAMP8 mice compared with the aged-matched SAMR1 (both in relative terms and in absolute numbers: *P*<0.01), whereas B2 cells were similar in both aged mice. Besides, the CD19 intensity (MFI) in B1REL cells and B2 cells from aged SAMP8 is higher compared to that from SAMR1 samples (CD19 MFI from B1REL: 23 081±278 and 11 000±384; from B2: 16 216±715 and 8541±201, for aged SAMP8 and SAMR1, respectively; mean±S.E.M.: *n*=5; ***P<0.001). MZ and ABC cells were identified on the basis of the CD21/CD23 phenotype on electronically gated B2 cells (Figure 1b). On aged SAMP8 mice compared to SAMR1 samples, MZ B-cells were diminished, whereas ABC cells were significantly increased (Figure 1c).

**Figure 2:**
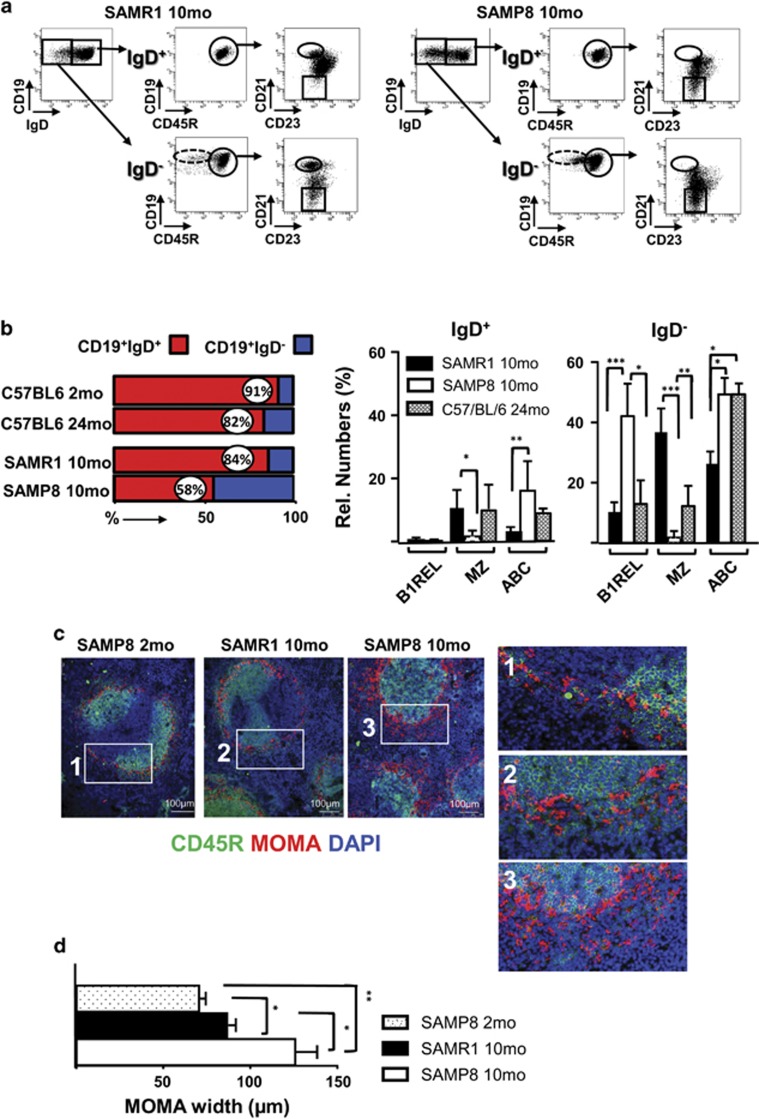
Phenotypic features of B lymphocytes and primary follicle organization in spleens from SAMP8 mice. The phenotype of the B1REL, B2, MZ and ABC B-cell subsets identified in the spleen of 10-month-old SAMR1 and SAMP8 mice, and the follicle distribution found in 2- and 10-month-old SAMP8 and SAMR1 mice, are shown. (**a**) Flow cytometry analyses were performed on spleen samples stained as in Figure 1b and with anti-IgD-FITC. Shown are representative dot-plots of the staining obtained from 10-month-old SAMR1 and SAMP8 samples. The left plot in each case, shows the distribution of IgD expression on gated CD19^+^ cells, identifying CD19^+^IgD^+^ and CD19^+^IgD^−^ subpopulations. The gates drawn inside the middle and right plots define, as in Figure 1b, the B1REL, B2,MZ and ABC cell subsets obtained from IgD^+^ cells (upper plots) and IgD^−^ cells (bottom plots). Fluorescence scales are logarithmic. (**b**) Left, the bar graph shows the distribution (as percentage of CD19^+^ cells) of IgD^+^ (red) and IgD^−^ (blue) cell subsets on 10-month SAMR1 (R1), and SAMP8 (P8) mice and on 24-month C57BL/6. Right column graphs, quantification of the indicated cell subsets as percentages of CD19^+^IgD^+^ and CD19^+^IgD^−^cells. Data are the mean±S.E.M. (*n*=7).Comparisons were made with a two-tailed Student’s *t*-test: *P<0.05; **P<0.01; ***P<0.001. (**c**) Immunofluorescence studies on splenic sections from the indicated samples. Three-color staining was performed using biotinylated MOMA-1 revealed with Cy3-tyramide (red), Cy5-labeled CD45R (green) and DAPI (blue). Brightness and contrast were adjusted using Photoshop version 7.0 (Adobe Systems Inc., San Jose, CA, USA) for display. Representative photomicrographs from each strain are shown. The boxes drawn in white define the regions amplified in the right, showing the MOMA band around primary follicles. Scale bars=100 *μ*m. (**d**) The graph bar represents the quantification of the MOMA band width (*μ*m) surrounding primary follicles. The band width was calculated on 7–10 different follicles per sample, performing four different measurements on each follicle. Samples from three different animals were analyzed in each case by two different investigators. The measurements of MOMA-1 band width were performed using the Leica application suite version 2.6.3 (Leica, Wetzlar, Germany). Data are the mean±S.E.M. Comparisons were made with a two-tailed Student *t*-test: *P<0.05; **P<0.01

It has been shown that naive B cells (IgD^+^) in spleen progressively decline in aged individuals, and this is accompanied by an accumulation of memory B cells (memBC) and plasma cells (PC) of restricted diversity.^33, 34^ In agreement with this, 24-month-old C57BL/6 mice had an important reduction of naive IgD^+^ B cells compared with the 2-month-old mice (Figure 2b and Supplementary Figure S2). This happened more profoundly in aged SAMP8 compared to SAMR1 mice (Figures 2a and b). A detailed phenotypic characterization showed that nearly all B1REL cells are IgD^−^ in samples from the three strains analyzed (Figure 2b), as are most of the ABCs and the MZ B cells (Figures 2a and b).

### Disrupted primary follicle structure in the spleen of SAMP8 mice and apoptosis/proliferation balance of ABC and B1REL cells

**Figure 3:**
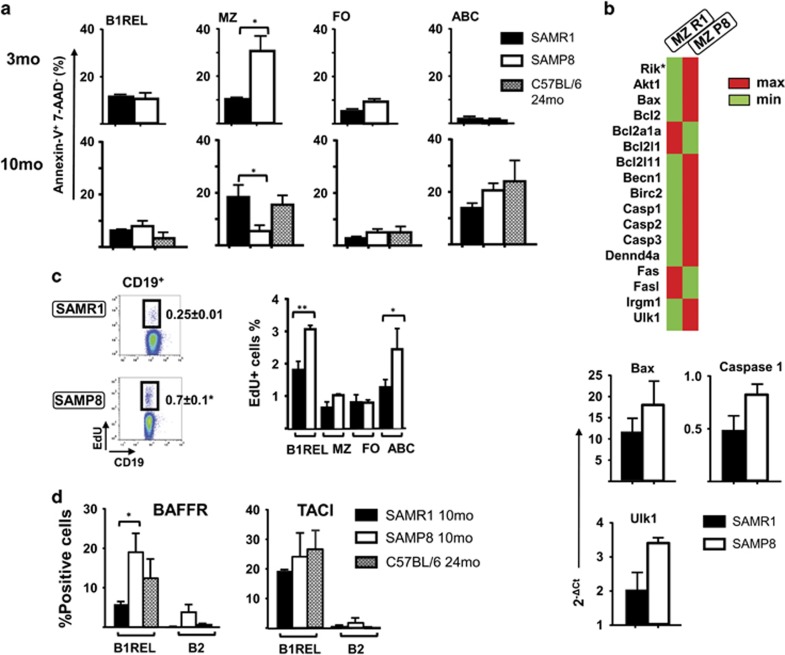
Apoptosis and proliferation in SAMP8 B-cell subpopulations. Splenocytes from 3- and 10-month-old of SAMR1, SAMP8 mice, and from 24-month-old C56BL/6, were stained as indicated in Figure 1b, and B1REL cells, B2 cells, MZ cells and ABCs were identified by flow cytometry. Apoptotic Annexin-V^+^ cells and proliferating EdU^+^ cells in the B-cell subsets were detected as indicated in the ‘Materials and Methods’ section. (**a**) Quantification (relative numbers) of the Annexin-V^+^7-AAD^−^ cells in each cell subset (mean±S.E.M., *n*=3). Comparisons were performed using a two-tailed Student *t*-test: *P<0.05. (**b**) Above, results of the cell death RT-qPCR array comparing MZ samples at 3-month-old from SAMP8 and SAMR1 mice are displayed. The heat map provides a visualization of the fold changes in expression between both groups for the selected genes in the array. Shown in red and green are genes with increased and reduced expression, respectively. *9430015G10 Rik. Below, RT-qPCR analyses for validation of Bax, Caspase 1 and Ulk1 genes as described in ‘Materials and Methods’, and expressed as the 2^−Δ*C*T^, relative to that of the GA3PDH transcripts. (**c**) Spleen cells from mice injected i.p. with EdU were analyzed after 16 h for the presence of EdU^+^ on gated CD19^+^ B cells. The left dot-plots show representative results from 10-month-old SAMR1 and SAMP8 mice. Fluorescence scales are logarithmic. The right-hand columns show EdU^+^ cell frequency in the indicated B cell compartments (means±S.E.M., *n*=3). (**d**) Staining with anti-CD19-Violet-421, anti-CD45R-FITC and anti-BAFFR-PE or anti-TACI-PE was performed to identify the expression of BAFFR and TACI on electronically gated B1REL cells. The graphs display the frequencies of B1REL cells expressing BAFFR or TACI. Data are means±S.E.M. (*n*=3). Comparisons were made with the unpaired two-tailed Student’s *t*-test: *P<0.05; **P<0.01

We wondered whether the marked changes observed in the numbers of different B cells might reflect an altered follicle organization. To directly visualize the MZ in aged mice, we performed immunofluorescence studies using the MOMA-1 mAb as a marker of metallophilic macrophages (Figure 2c). Compared with the 2-month-old SAMP8 mice as control, old SAMP8 and SAMR1 animals showed an altered and thicker MOMA band (Figure 2d), as described for aged BALB/c.^35^ The disrupted MOMA band observed in aged SAMP8 mice may affect the distribution of B cells, perhaps altering their viability, and may thus reflect differences in apoptosis or proliferative status of the cells. We studied apoptosis in B-cell subsets at 3- and 10-month-old on SAMP8 and SAMR1 mice, and from 24-month-old C57BL/6 mice, using the Annexin-V detection (Immunostep, Salamanca, Spain) technique (identifying early apoptotic cells as Annexin-V^+^7-AAD^−^), and proliferation *in vivo* by detecting EdU-incorporation. Apoptosis levels in MZ from 3-month-old SAMP8 mice were greater than those found in SAMR1 samples and for the rest of the B-cells subsets. Accordingly, MZ cells from SAMP8 mice showed an increase of transcripts related with cell death and autophagy (Figures 3a and b) when compared with the SAMR1 samples. In samples from 10-month-old mice, apoptosis levels on ABCs and B1REL cells were similar, with the higher rates in ABCs and lowest in B1REL cells (Figure 3a). The residual MZ cells found in aged SAMP8 mice, showed lower apoptosis than MZ cells from aged SAMR1 and 24-month-old C57BL/6 mice, due to the apoptotic mechanisms operating earlier in life in SAMP8 mice (Figure 3a), which led to the impoverished subset of these cells. On the other hand, more ABCs and B1REL cells from old SAMP8 mice incorporated EdU than these cell subsets from age-matched SAMR1 mice, indicative of a higher proliferative status (Figure 3c), in agreement with results on B1REL cells from BALB/c mice.^25^

Previous results from our laboratory showed that B1REL cells expressed TACI and BAFFR,^24^ receptors for survival factors such as BAFF (B cell-activating factor) and APRIL (A proliferation-inducing ligand or tumor necrosis factor ligand superfamily member 13). Therefore, we have checked for the expression of TACI and BAFFR, in B1REL cells from aged SAMP8, SAMR1 and C57BL/6 mice (Figure 3d). An enhanced expression of BAFFR was observed on B1REL samples from SAMP8 mice, a fact that may contribute to the accumulation of B1REL cells observed on aged SAMP8 mice. By contrast, TACI^+^ B1REL cells were not increased in samples from SAMP8 mice.

Overall, these data prompts us to conclude that the deconstructed primary follicles in aged SAMP8 mice, accompanied by higher proliferation rates of B1REL cells and ABCs, and the higher BAFFR expression on B1REL cells, may contribute to the accumulation of ABCs and B1REL cells and to the reduction of MZ cells.

### Impaired terminal differentiation of B cells in aged SAMP8 mice

**Figure 4:**
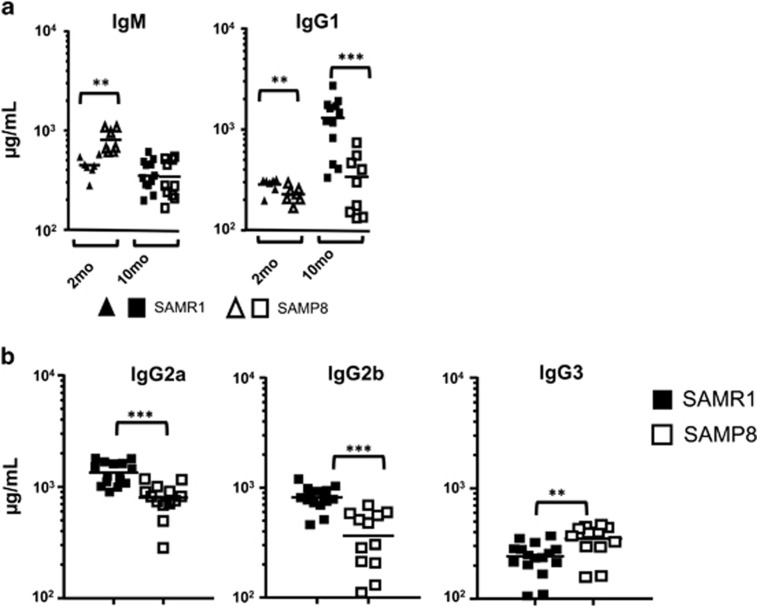
Immunoglobulin isotypes in serum from SAMR1 and SAMP8 mice determined by ELISA. (**a**) Serum IgM and IgG1 from 2- and 10-month-old SAMR1 and SAMP8 mice. (**b**) IgG2a, IgG2b and IgG3 isotypes were quantified on sera from 10-month-old SAMP8 and SAMR1 mice. Data are measurements of individual mice. Means are indicated by the horizontal lines. Comparisons were made with the unpaired two-tailed Student’s *t*-test: **P<0.01; ***P<0.001

**Figure 5:**
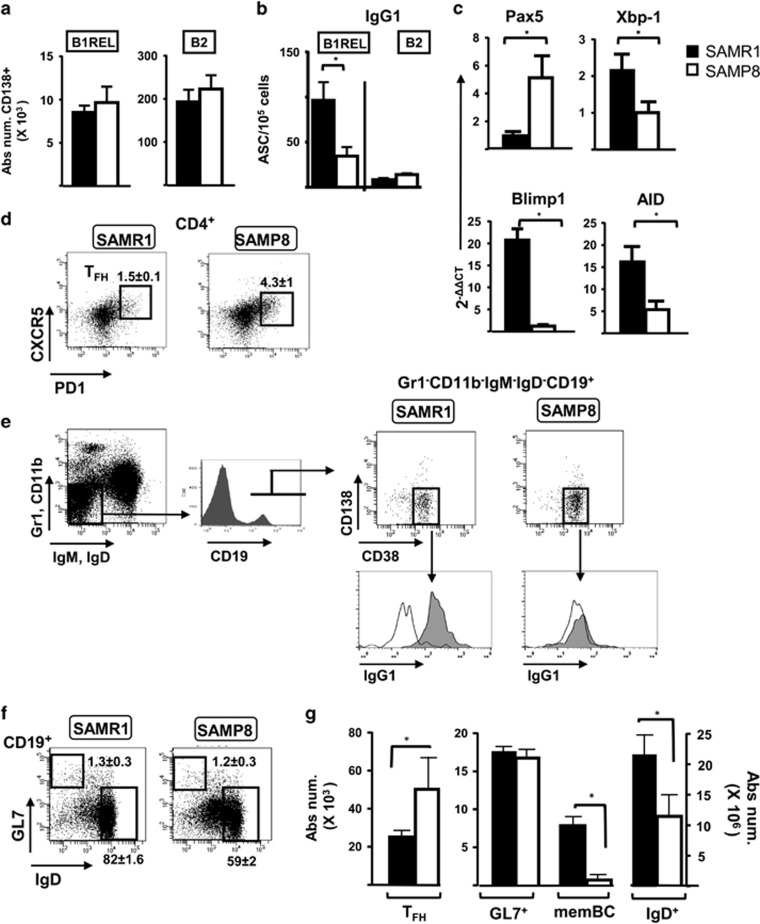
Plasma cell B cell maturation and distribution of the memory B-cell compartments in aged SAMP8 mice. (**a**) Cells were stained with anti-CD19-Violet-421, anti-CD45R-PE and anti-CD138-APC. Absolute numbers of CD138^+^plasmablasts were calculated from flow cytometry as in Figure 1c. Data are mean±S.E.M., (*n*=4). (**b**) IgG1-specific ELISPOT analysis (see ‘Materials and Methods’) of purified samples of B1REL and B2 cells obtained from aged SAMR1 and SAMP8 mice. The numbers of IgG1- antibody secreting cells (ASCs) are shown. Data are mean±S.E.M., (*n*=4). (**c**) RT-qPCR analyses were performed for Pax5, Xbp-1, Blimp1 and AID transcript expression in purified B1REL cells from 10-month-old SAMR1 and SAMP8 mice (see ‘Materials and Methods’). The Bio-Rad CFX Manager software (Bio-Rad, Hercules, CA, USA) was used to calculate the *C*_T_ of each reaction. The amount of specific transcripts in each cDNA sample was determined as the 2^−ΔΔ*C*T^, relative to that of the HPRT transcripts and normalized to those obtained for 2-month-old BALB/c samples used as reference. Data are mean±S.E.M. (*n*=6–10 performed in duplicates). (**d–f**) Flow cytometry analyses 10-month-old SAMR1 and SAMP8 mice spleen samples. Representative dot-plots are shown for each mouse. Numbers inside the plots are frequencies of each population (mean±S.E.M.: *n*=4 for T_FH_ cells and for GL7^+^ and IgD+ cells, and *n*=3 for memBC). Fluorescence scales are logarithmic. (**d**) Staining was performed with anti-CD4-APC, anti-CXCR5-Violet-421 and anti-PD1-biotynilated and revealed with streptavidin-PE-Cy7. T_FH_ cells were gated as CD4^+^CXCR5^+^PD1^+^ cells (boxes). (**e**) MemBC were determined as CD11b^−^Gr1^−^CD138^−^IgM^−^IgD^−^CD19^+^CD38^+^IgG1^+^ by using anti-CD11b- and anti-Gr1-PE-Cy7, anti-CD138-PE, anti-IgM- and anti-IgD-FITC, anti-CD19-Violet-421, anti-CD38-APC and rat anti-mouse IgG1-biotynilated (clone A85-1, rat IgG1/k). The FMO control was a biotinylated rat IgG1/*k* (clone R3-34). Biotynilated Abs were revealed with streptavidin-APC-Cy7. The histograms display the IgG1^+^ cells among the CD138^−^CD38^+^ cells (empty, FMO isotype control; filled gray, IgG1^+^ cells). (**f**) Staining was performed with anti-CD19-Violet-421, anti-GL7-PE and anti-IgD-FITC, to determine GL7^+^ cells and naive IgD^+^ cells on gated CD19 cells. (**g**) Bar graph shows the absolute number/spleen of T_FH_, GL7^+^, memBC and naive B cells (right *Y* scale). These absolute numbers were calculated from the frequencies of each population. Shown are the mean±S.E.M. (*n*=4 and 3 for memBC). The group comparisons were made using the unpaired two-tailed Student’s *t*-test: *P<0.05

To trace whether these changes led to any functional alteration, we first quantified the IgM, IgG1, IgG2a, IgG2b, IgG3 and IgA levels in sera of 2- and 10-month-old SAMP8 and SAMR1 mice. IgM levels, which were higher in 2-month-old SAMP8 than in SAMR1 sera, diminished in sera from aged SAMP8 mice, becoming then similar in both strains. On the other hand, an enhancement of IgG1 levels was detected in sera from aged SAMR1 mice with respect to 2-month-old mice, but not on aged SAMP8 mice (Figure 4a), which had lower IgG1, IgG2a and IgG2b levels, but not IgG3 (Figure 4). IgA levels were found to be very low in sera from both 10-month-old SAMP8 and SAMR1 mice (24.76±1.4 *μ*g/ml and 22.34±1.5 *μ*g/ml, respectively; *n*=11), values that were much lower than those found in C57BL/6 mice sera (314±1.5 *μ*g/ml, *n*=3), in agreement to the described impairment to secrete this IgA isotype for AKR/J background mice (as are SAM strains).^36^ We next quantified PC numbers by analyzing CD138 expression in electronically selected B1REL and B2 cells. The total number of CD138^+^ PCs in these cell subsets was similar in both strains (Figure 5a), which may indicate a selective defect in the secretion of IgG-switched Igs in SAMP8 mice. Previous results from our group showed that B1REL cells from 2-month-old BALB/c mice were poised to spontaneously secrete IgG1 and contained high numbers of IgG1-ASC. Therefore, we quantified by ELISPOT assays the number of IgG1-ASCs among purified B1REL and B2 cells from aged SAMP8 and SAMR1 mice (Figure 5b). The results showed a strong reduction in IgG1-ASC among B1REL cells from SAMP8 mice compared with the SAMR1 mice.

The balance of several transcription factors (an increase in Xbp-1 and Blimp1, and a decrease of Pax5, which also controls CD19 expression) regulates terminal B cell differentiation to PCs. We assessed by qRT-PCR the Pax5, Xbp-1, Blimp1 and AID mRNA expression in samples of B1REL cells from aged SAMR1 and SAMP8 mice (Figure 5c). Accordingly with the low IgG1-ASC and serum IgG1, IgG2a and IgG2b levels found in aged SAMP8 mice, a weaker Xbp-1, Blimp1 and AID expression signal was detected in B1REL cells. By contrast, an increase in Pax5 expression was observed, in agreement with the above mentioned high CD19 MFI brightness observed in B1REL cells from SAMP8-aged mice.

Immunological memory is enriched in elderly individuals as a consequence of multiple Ag encounters throughout life.^37^ During the germinal center (GC) reaction, activated B cells undergo affinity maturation, class-switch recombination and differentiation through signals mediated by T follicular helper cells (T_FH_). We have determined the numbers of T_FH_ cells (CD4^+^PD1^+^CXCR5^+^), IgG1-memBCs (CD11b^−^Gr1^−^CD138^−^IgM^−^IgD^−^CD19^+^CD38^+^IgG1^+^), GC GL7^+^ B cells (CD19^+^CD45R^+^CD38^+^GL7^+^) and naive CD19^+^IgD^+^ cells on samples from aged SAMP8 and SAMR1 mice (Figures 5d–g). Splenic samples from aged SAMP8 mice had increased T_FH_ cells. By contrast, IgG1-switched memBC were significantly diminished on these samples, in comparison with those from SAMR1 mice, as happened for naive CD19^+^IgD^+^ B cells (Figures 2 and 5g). GC GL7^+^ B cells remained at similar levels in both animals. These data led us to conclude that in the primary GC in SAMP8 mice, aging causes a significant reduction in the IgG1-switching process of terminal differentiation, involving IgG1-PCs and IgG1-memBCs, and further resulting in the accumulation of T_FH_ in samples from SAMP8 mice.

### Immunoglobulin repertoires in aged SAMP8 and SAMR1 mice

IgH repertoires were studied in FACS-purified B1REL and B2 cells from aged SAMP8 and SAMR1 mice, by analyzing the VDJ_H_-C_*μ*_ and VDJ_H_-C_*γ*1_ joints expressed in these samples (Supplementary Figures S3 and S4). The type of V_H_ to J_H_ rearrangements in the sequences is represented in circle graphs (Supplementary Figure S3a), where the number and thickness of the ribbons inside circles reflects the pattern and frequency of usage of V_H_-J_H_ joints. In general, the VDJ_H_-IgG1 repertoire in B cell populations from aged SAMP8/SAMR1 mice has low diversity. In addition, the V_H_ and D_H_ usage in sequences from B1REL cells of aged SAMR1/SAMP8 mice differed (Supplementary Figure S4), as did the amino-acid (AA) composition in the CDR-H3 regions in the IgG1-B1REL sequences, which was prone to adopt a hydrophobic profile. The CDR-H3 region length (in SAMP8 samples) and the mutation rate (in SAMR1 and SAMP8 samples) of the IgG1 sequences was higher than that of the IgM sequences, although Ag selection gave moderate levels of this variable, denoting that most of the mutations found were silent or not located at CDRs. In summary, the reduction in diversity, the increase in length and mutation rates, and the hydrophobic AA usage in the CDR-H3 region of the V_H_ repertoire in these B cell populations from aged SAMP8/SAMR1 mice, reflects an aged repertoire, as expected.

### Poor responsiveness of B-cell subsets from aged SAMP8 mice to LPS stimulation

**Figure 6:**
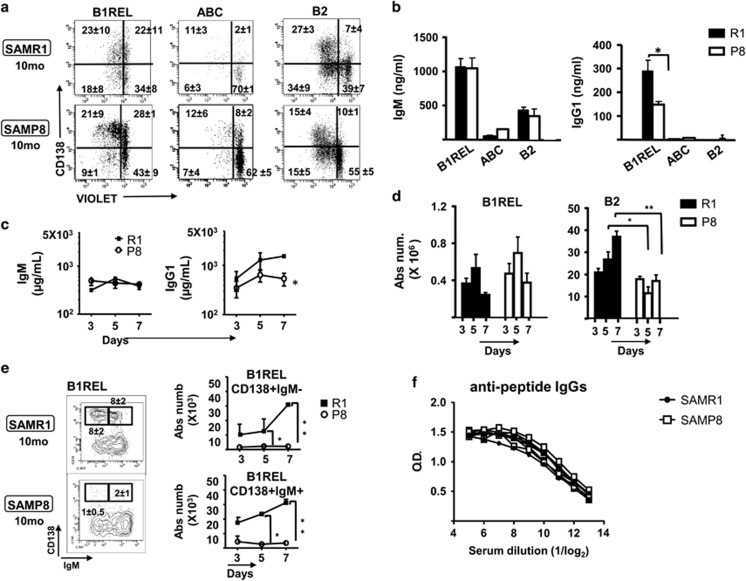
*In vitro* and *in vivo* responses of B-cell subsets from SAMP8 mice stimulated with LPS (**a**) and (**b**). The indicated B-cell subsets identified as in Figure 1b from spleens of 10-month-old SAMP8 and SAMR1 mice were FACS-purified, and then labeled with the CellTrace Violet kit before culturing for 72 h in the presence or absence of LPS (see Materials and Methods). After culture, the cells were washed and stained to detect CD138 and the incorporated violet dye by flow cytometry. (**a**) Representative results from B1REL, ABC and B2 cells after LPS stimulation are shown. Fluorescence scales are logarithmic. The numbers in the plots are the frequency in each quadrant. Data are means±S.E.M. (*n*=3). (**b**) The IgM and IgG1 secreted into the culture medium after 72 h were determined by ELISA. Data are means±S.E.M. (*n*=3). Comparisons were made using a two-tailed Student’s *t*-test: *P<0.05. (**c–e**) 10-month-old SAMR1 and SAMP8 mice were injected i.p. with LPS and after 3, 5 and 7 days serum and cell suspensions from spleens were obtained. (**c**) The IgM and IgG1 levels in sera were determined by ELISA. (**d**) Absolute numbers of B1REL and B2 cells (determined as in Figure 1c) are shown. (**e**) Left, representative contour plots of splenic cells stained with anti-CD19-PE, anti-CD45R-FITC, anti-IgM-PE-Cy7 and anti-CD138-byotinilated revealed with streptavidin-APC at day 7 post-injection. B1REL cells were identified as in Figure 1b, and electronically gated to show the distribution of CD138 and IgM on them. The gates within the plots identify CD138^+^IgM^−^ and CD138^+^IgM^+^cells. The numbers are the frequencies of these cells. Right, absolute numbers of CD138^+^IgM^−^ and CD138^+^IgM^+^ B1REL cells were calculated at the indicated times after LPS injection. The data were obtained from the frequencies of each cell population (means±S.E.M., *n*=3–4). Comparisons were made with the unpaired two-tailed Student’s *t*-test: *P<0.05; **P<0.01. (**f**) Titration of anti-peptide-specific IgGs after *in vivo* immunizations with peptide-OVA. Aged SAMP8 (*n*=5) and SAMR1 (*n*=3) were immunized with peptide-OVA (see ‘Materials and Methods’), and the amounts of IgG specific for the peptide were determined by indirect ELISA in serial dilutions of the sera. Shown are the data for each individual mouse

To further define the implications of the splenic B-lymphoid distribution in aged SAMP8 samples, we isolated B1REL, ABCs and B2 cells, and studied their proliferation in response to LPS stimulation *in vitro* using the CellTrace Violet dye (Invitrogen-ThermoFisher Scientific, Waltham, MA, USA) (Figure 6a). MZ cells were nearly absent in samples from aged SAMP8 mice and we could not obtain enough purified cells to perform *in vitro* cultures. LPS stimulation of B1REL cells isolated from aged SAMP8 and SAMR1 mice induced a weak *in vitro* proliferative and differentiation response, determined by the dilution of the Violet tracer and the induction of CD138. The ABCs isolated from aged SAMP8 and SAMR1 animals were not responsive after the challenge with LPS, as they are a refractory cell subset as described.^17^ B2 cells from aged SAMP8 mice proliferated less than that from aged SAMR1 mice (*P<*0.05). Finally, the supernatants from B1REL cell cultures in aged SAMP8 mice had reduced IgG1 levels compared to those from aged SAMR1 cultures, whereas equivalent amounts of IgM were found on supernatants from B1REL, B2 cells and ABC cultures of cells from SAMP8 and SAMR1 mice (Figure 6b). These data support the idea of functional intrinsic and differential defects in the different B-cell subsets in aged SAMP8 mice.

The loss of MZ B cells together with the augmentation of B1REL and ABCs, points out to a scenery where the rapid non-specific responses, usually related with MZ activation, could be altered. As LPS drives the activation and differentiation of MZ B cells and B1REL cells^25, 38, 39^ *in vivo* T cell-independent stimulation with LPS was studied by measuring IgM and IgG1 secretion in the serum (Figure 6c). A significant decrease in IgG1 was evident in aged SAMP8 samples that may reflect a poor activation of the B1REL compartment in these mice, despite its elevated numbers. We also monitored the total number of B1REL and B2 cells present in the spleen at 3–7 days after LPS immunization. B1REL and B2 cells increased in 2-month-old BALB/c mice after 5 and 7 days, indicating that B1REL cells actively responded in this model of immunization (Supplementary Figure S5). In spleen samples from aged SAMP8 and SAMR1 mice, there was an increase in B1REL cells at 3 and 5 days after LPS-activation, which was not maintained at 7 days (Figure 6d). Furthermore, there was virtually no CD138 induction in B1REL cells from aged SAMP8 mice, irrespective of IgM expression, compared with the aged SAMR1 samples (Figure 6e). To test whether this impairment of polyclonal humoral responses was extensive to antigen-specific T-dependent activations, we performed an immunization schedule with peptide-OVA, and checked for anti-peptide-specific IgG in the sera 1 week after the boost. The results show (Figure 6f) that aged SAMP8 and SAMR1 mice produced similar levels of peptide-specific IgG, limiting the defect found on these aged SAMP8 animals to T-independent responses.

## Discussion

A decline in the effector functions of the immune system during aging and the decreased effectiveness of the antibodies produced in the humoral response of elder individuals,^8^ represent major health issues in developed countries. These inefficient humoral responses in aged individuals can be attributed to different causes, such as reduced diversity of the Ig repertoire, a lower affinity of the Abs generated or limited levels of certain Ig isotypes.^9, 10, 40, 41^ Elderly humans accumulate B cells in peripheral blood, which is enriched in memBC, and they produce fewer naive B cells with long CDR-H3 regions.^10, 30^ In addition, an imbalance in certain B-cell compartments in SAMP8-aged mice, as that found here for the ABCs, or B1REL and MZ cells, may also contribute to unproductive humoral responses. A progressive decline with age in the *in vitro* and *in vivo* humoral responses has been reported in SAMP8 mice.^42, 43^ Here we have addressed the role of B-cell subpopulations in these defective humoral responses.

We failed to find major changes in the distribution of total T, B and myeloid cells in the bone marrow and other lymphoid territories, although we did detect a relative increase in B cells accompanied by a decrease in T cells in the spleen. A detailed phenotypic characterization of the different B-cell subsets revealed significant changes in the spleen, specifically in the strong accumulation of B1REL cells and ABCs, with the marked decrease in MZ and naive B cells. Furthermore, the disorganization of the MOMA band in aged SAMP8 mice highlights changes in primary follicle organization, which may affect the numbers of MZ B cells detected in this context. Progression of GC reactions on aged SAMP8 mice samples was inefficient, in terms of the decline detected on IgG1-switched memBC that was accompanied by an accumulation of T_FH_ cells.

Aging induces changes in serum Ig composition in association with the reduced capacity for isotype switching.^44, 45^ The data presented here demonstrate a lower serum IgG1, IgG2a, and IgG2b content in aged SAMP8, confirming previous results.^42^ Accordingly, B cell maturation to PCs and isotype switching programs (Xbp-1, Blimp1 and AID) are profoundly affected in aged SAMP8 mice. As Blimp1 promotes cell cycle arrest,^46^ the low levels of this transcription factor in B1REL cells from aged SAMP8 mice may favor cell cycle activation and proliferation, resulting in the accumulation of this cell subset.

The V_H_ repertoires of the IgM and IgG1 transcripts isolated from B1REL and B2 cells from aged SAMP8 mice, reflect a decline in diversity, as described in humans, and this translates into a narrow Ig repertoire in response to pathogens. Interestingly, we found that the mutation rates and the Ag-driven selection of the IgH rearrangements were comparable to those found for splenic B cells from adult mice,^47^ possibly reflecting the tonic activation necessary to maintain the homeostatic environment. The length and hydrophobic profile in the CDR-H3 AA composition^48^ may suggest evidence of auto-antibodies, which were described previously on aged SAMP mice.^32^

When the response of isolated B-cell subsets to *in vitro* LPS activation was quantified, both the proliferative and differentiation potential of B1REL cells and conventional B2 cells was dampened in aged SAMP8 mice, and there was no response of ABCs to LPS activation, suggesting the functional exhaustion of the ABCs subset. *In vivo* LPS injection on aged SAMR1/P8 mice results in weaker IgG1 production in aged SAMP8 mice. However, *in vivo* antigen-specific T-dependent responses were similar in both aged mice, a result that limits the defective humoral responses of aged SAMP8 to the rapid responses involving MZ cells that is circumvented in T cell-dependent responses involving B2 and T cells.

We have found that B-cell subsets in peripheral organs (e.g., the spleen) are affected in aged SAMP8 mice differently than in age-matched SAMR1 or C57BL/6 mice. First, the distribution of B cell subpopulations (MZ cells, B1REL cells, ABCs and naive B cells) is altered, and there is a notable impairment on the GC formation and the terminal differentiation process of B cells to switched-PCs or to memBC generation. Second, the activation of different B-cell subsets was dampened in aged SAMP8 mice, and it was inhibited in the case of ABCs. Third, the humoral IgG1 response derived from T-independent activation was profoundly altered in aged SAMP8 mice. These striking changes in B-cell distribution and activation in immunized SAMP8 mice may have important consequences for the humoral response upon different antigenic encounters, provoking a defective IgG1 secretion in a homeostatic context and in the initial MZ response. Altogether, these data prompt us to propose this mouse model as an appropriate system to explore future immune therapies to recover a stronger and more appropriate humoral response of potential benefit to elder humans.

## Materials and methods

### Mice and *in vivo* immunizations

C57BL/6, SAMP8, SAMR1 mice were bred and maintained in the SPF animal facilities at the Instituto de Salud Carlos III (ISCIII, Madrid, Spain). The SAMP8 and SAMR1 mice were kindly provided by Dr. Mercè Pallaàs from the Universitat de Barcelona. For *in vivo* LPS injections, mice were treated intraperitoneally (i.p.) with 100 *μ*g of lipopolysaccharide (LPS, Sigma-Aldrich, St Louis, MO, USA). Serum samples were obtained 3, 5 and 7 days after immunization, and the Ig titer was tested. For *in vivo* peptide-OVA immunizations the mice were injected i.p. with 25 *μ*g/0.1 ml emulsified in CFA, and 15 days later with of 12.5 *μ*g/0.1 ml emulsified in IFA. Seven days after the boost, animals were bled to determine the titer of the peptide-specific antibody response. All animal studies were approved by the Institutional Review Board at the ISCIII and carried out in accordance with EU and National guidelines, PROEX (015/14, 080/14, 110/15 and 278/14).

### Flow cytometry and cell sorting

Single cell suspensions were prepared in staining buffer (2.5% Foetal Calf’s Serum) in Dulbecco’s Phosphate buffered saline (BioWhittaker, Lonza Group, Visp, Switzerland) and non-specific binding was blocked with Fc-Block (BD Biosciences, San Jose, CA, USA). Cell subpopulations identified in this report were defined by flow cytometry techniques with the antibodies and secondary reagents listed in Supplementary Table S1. Doublets were discriminated using the FSC-H *versus* FSC-W strategy, and cell viability was assessed by staining with by propidium iodide (PI) unless indicated. The cells were analyzed on a FACSCanto-I and a LSR Fortessa X-20 (BD Biosciences) cytometer, using the FlowJo v6.3.4 (TreeStar, Ashland, OR, USA) and DIVA v8.0 (BD Biosciences) software packages, and they were FACS-purified (over 95% purity) using a FACSAria-I (BD Biosciences) cell sorter.

### Annexin-V assay of apoptosis

Cells were analyzed using the Annexin-V-PE Apoptosis Detection kit (Immunostep, Salamanca, Spain), following the manufacturer’s instructions. Splenocytes were resuspended at 1 × 10^6^/ml and surface stained as indicated. The evaluation of apoptosis was based on Annexin-V staining of cells that did not incorporate 7-AAD.

### *In vivo* cell proliferation assay

Proliferation *in vivo* was assessed by EdU (5-ethynyl-2′-deoxyuridine) incorporation into DNA. Mice were injected intraperitoneally with 1 mg EdU (Molecular Probes, Carlsbad, CA, USA) and killed 16 h later.

### ELISA

Sera collected from SAMR1 and SAMP8 mice were analyzed as described previously.^49^ Standard curves for each Ig isotype were generated using purified myeloma proteins or antibodies: IgM (clone G155-228, BD Biosciences), IgG1 (clone 4.19, in-house),^50^ IgG2a (clone PK136, in-house), IgG2b (clone Y.3, in-house), IgG3 (clone MG3-3S, Biolegend, San Diego, CA, USA). The Ig concentrations were calculated by using the Graph Pad Prism 5.0 software. After *in vivo* peptide-OVA immunization, anti-peptide-specific IgG titers in sera were determined by indirect ELISA in Nunc 96-microplate wells (Maxi Sorp, 442404, Nunc-Thermo Fisher Scientific). Plates were coated (1 h, 37 °C) with 50 *μ*l of peptide (10 *μ*g/ml PBS); plates were then washed, blocked, incubated with sera (twofold serially diluted in PBS-Tween20/BSA, starting from 1:10), and revealed with goat anti-mouse IgGs (SouthernBiotech, Birmingham, AL, USA), as described.^49^ The 18-amino-acid peptide is part of the sequence of the H toxin of *S. aureus*. Coupling agent: Sulfo-SMCC (succinimidyl 4-(*N*-maleimidomethyl)cyclohexane-1-carboxylate).

### ELISPOT

Ig-secreting cells were quantified in Ig-specific assays as described previously.^24^ In brief, purified goat anti-mouse total Ig (10 *μ*g/ml: SouthernBiotech) was used to coat 96-well plates overnight at 4°C. After blockade with 1% gelatin in PBS, serial dilutions of the purified cell populations were cultured overnight at 37 °C in triplicates. Cells were incubated with either a biotinylated goat anti-mouse IgM or with a biotinylated goat anti-mouse IgG1 (Southern Biotechnology Associates), and then revealed with streptavidin-conjugated alkaline phosphatase (1 h, 37 °C: SouthernBiotech) followed by 5-bromo-4-chloro-3-indolyl-phosphate in 4% low-melt agarose (Sigma-Aldrich, St. Louis, MO, USA).

### Confocal studies

Slides from frozen spleen sections were examined under a Leica DMI3000 confocal microscope (Leica, Wetzlar, Germany) using × 20 and × 40 oil immersion lenses. Immunofluorescence was performed as described.^51^ In brief, tissue sections were treated with NH_4_Cl (50 mM) before blocking them with 10% mouse serum and with the Biotin Blocking System (DakoCytomation, Glostrup, DK).The sections were then incubated with an FITC-labeled anti-CD45R and a biotinylated anti-MOMA-1 (CD169, Siglec-1) mAb. The tyramide signal-amplification system (PerkinElmer, Waltman, MA, USA) was used to visualize the binding of the biotinylated anti-MOMA-1 and an Alexa Fluor 488 anti-FITC to amplify the FITC anti-CD45R. Endogenous peroxidase activity was quenched with hydrogen peroxidase and the nuclei were counterstained with DAPI.

### Quantitative real-time PCR, cloning and V_H_ region sequencing

Total RNA was extracted (Leica, Wetzlar, Germany) using the RNeasy Micro kit method (Qiagen, Hilden, Germany) from purified splenic B1REL and B2 cells from aged SAMR1 and SAMP8 mice and from 2-month-old BALB/c mice as a control. Oligo (dT)-primed cDNA samples were prepared in 25 *μ*l with avian myeloblastosis virus reverse transcriptase as described previously,^49^ and 1 *μ*l of each cDNA was used for further PCR amplifications. RT-qPCR was performed as indicated elsewhere^52^ on a CFX96 Real-Time System using the SsoFastSupermixEvaGreen (Bio-Rad, Hercules, CA, USA). Bio-Rad CFX Manager software was used to calculate the *C*_T_ of each reaction and the relative amount of specific cDNA in each sample relative to the expression of the *hypoxanthine phosphoribosyl transferase-1* gene (HPRT) was determined by the 2^−Δ*C*T^ method.^51^ The values were normalized by using a control sample from 2-month-old BALB/c mice for each transcript, and the results expressed as the 2^−ΔΔ*C*T^. The primers used for Pax5, Xbp-1, Blimp1, AID and HPRT were those described previously.^24, 47, 53^

### Cell death PCR arrays

Cell death PCR arrays were performed on RNA samples from sorted MZ cells as described^24, 25^ using a *RT*^2^ PreAMP Primer Mix and to *RT*^2^ Profiler PCR Array mouse cell death pathway Finder (SABiosciences-Qiagen, Hilden, Germany). Heat map data were generated using web-based software available on http://www.sabiosciences.com/dataanalysis.php. qPCR validation of Caspase1 and Ulk1 transcripts was performed using primers and PCR conditions described.^54, 55^ For Bax validation, the following primers were used; Bax-1: Forward 5′-ATCGAGCAGGGAGGATGGC-3′ Reverse: 5′-GAGCACCAGTTTGCTAGCAAAG-3′, and the same PCR conditions as described.^54, 55^

### V_H_ region sequencing

Detection of IgH VDJ-C rearrangements, cDNA preparations of purified B1REL and B2 cells were subjected to PCR amplification with 1 U of FastStart DNA polymerase (Roche, Mannheim, Germany) in a PTC-200 DNA Engine cycler (Bio-Rad). Rearranged alleles were amplified, cloned and sequenced using IgH specific primers and PCR conditions described previously.^47^ The sequences analyzed in this study have been deposited in the Genebank database with the accession numbers JZ923098 to JZ923691. Alignments of the sequences obtained were studied using IgBLASTN (http://www.ncbi.nlm.nih.gov/igblast/) and the ImMunoGeneTics information system (http://www.imgt.org/HighV-QUEST/).^56^ Comparative sequence analysis between populations and the complementarity determining region of the antibody heavy chain (CDR-H3) amino-acid alignments were performed using the Immunoglobulin Analysis Tool software, IgAT.^57^ Circle graphs were obtained using the available software (http://circos.ca/).

### *In vitro* cultures of B lymphocytes

Proliferation assays on isolated B cell populations were performed using the CellTrace Violet kit (Invitrogen-Thermo Fisher Scientific) following the manufacturer's instructions. Purified B1REL, MZ, ABC and follicular lymphocytes were loaded with the Violet dye (20 min at room temperature in PBS 1 × 10^6^/ml cells). The cells were then plated in flat-bottomed 96-well culture plates (BD Biosciences) and cultured at 1 × 10^5^/100 *μ*l for 72 h at 37 °C and 5% CO2 in complete RPMI 1640 (10% heat-inactivated FCS, 2 mM L-glutamine, 1 mM pyruvate, 50 mM 2-ME, 10 mM HEPES and antibiotics) in the presence or absence of LPS (25 *μ*g/ml). After culture, the cells were washed and labeled with anti-CD138-APC using standard protocols,^25^ before being analyzed by flow cytometry.

### *In vivo* cell proliferation assay

In brief, splenocytes were obtained and the incorporated EdU was detected using the Click-iT Plus EdU-Alexa Fluor 647 Flow Cytometry Assay Kit (Molecular Probes), according to the manufacturer’s instructions. Briefly, 1 × 10^7^ cells/ml were submitted to Click-iT fixative and saponin-permeabilization procedures before adding the Click-iT reaction cocktail during 30 min at room temperature. Cell suspensions (2 × 10^6^ cells per tube) were stained with the mAbs indicated to define B1REL, MZ, ABC, and follicular B cells by flow cytometry.

### Statistical analysis

The data are presented as the means±S.E.M. Statistical analyses were performed with Prism 5.0 (Graph Pad Software Inc., La Jolla, CA, USA) software after testing the data distributions using the Kolmogorov–Smirnov, Shapiro–Wilk and D’Agostino–Pearson normality tests. Comparisons were performed with the two-tailed Student’s *t*-test and the *χ*^2^ test for contingency tables: *P<0.05, **P<0.01, *** *P*<0.001.

## Publisher’s Note

Springer Nature remains neutral with regard to jurisdictional claims in published maps and institutional affiliations.
