# Supplementary material for: Altered marginal zone and innate-like B cells in aged senescence-accelerated SAMP8 mice with defective IgG1 responses
Source: Cell Death Dis. 2017 Aug 17;8(8):e3000–. doi: 10.1038/cddis.2017.351 (PMC5596542; doi:10.1038/cddis.2017.351)
Supplement: Supplementary Figure S1 [file cddis2017351x1.pdf]

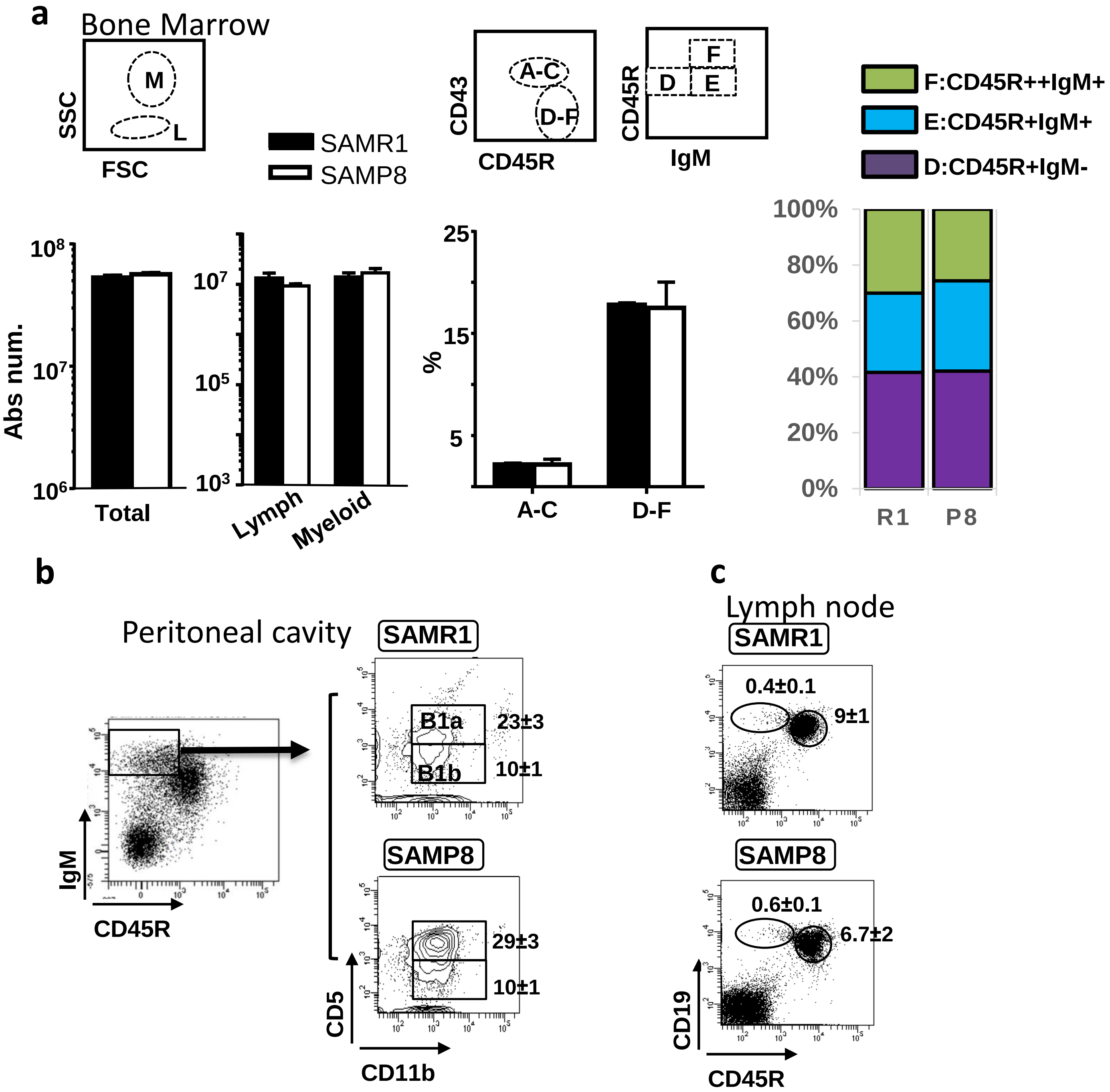

Figure S1: B-lymphocyte compartments of aged SAMR1 (10-month-old) and SAMP8 (10-month-old) mice from bone marrow (BM), peritoneal cavity (PWC) and lymph node (LN) by flow cytometry. (a) Bone marrow cells were obtained from the tibias and femurs and stained with anti-CD43 PE, anti-CD45R PE-Cy7 and anti-IgM APC-Cy7 in order to trace immature bone marrow B-cell progenitors (mean ± SEM: n = 3-4). Lymphoid, myeloid and total cellularity were calculated. B-cell lymphopoiesis were calculated on the basis of fractions A-C and D-F described by *R.R. Hardy et al (1993),* expressed as frequencies. (**b)** Peritoneal cells were stained with anti-CD19 PE, anti-IgM FITC, anti-CD45R PE-Cy7, anti-CD5 APC, and anti-CD11b APC-CY7. Cells were gated on the basis of CD19+IgM+CD45Rlo in order to trace B1a (CD5+, CD11b+), and B1b (CD5-, CD11b+). (**c**) Inguinal lymph nodes were processed and stained with anti-CD45R PE-Cy7 and anti-CD19 PE. B Lymphocytes were gated on CD19+cells. (mean ± SEM: n = 3-4).

Hardy R.R. 1993. The regulated expression of B lineage associated genes during B cell differentiation in bone marrow and fetal liver. *J. Exp. Med*. 178:951-60.
