# Supplementary material for: Altered marginal zone and innate-like B cells in aged senescence-accelerated SAMP8 mice with defective IgG1 responses
Source: Cell Death Dis. 2017 Aug 17;8(8):e3000–. doi: 10.1038/cddis.2017.351 (PMC5596542; doi:10.1038/cddis.2017.351)
Supplement: Supplementary Figure S2 [file cddis2017351x2.pdf]

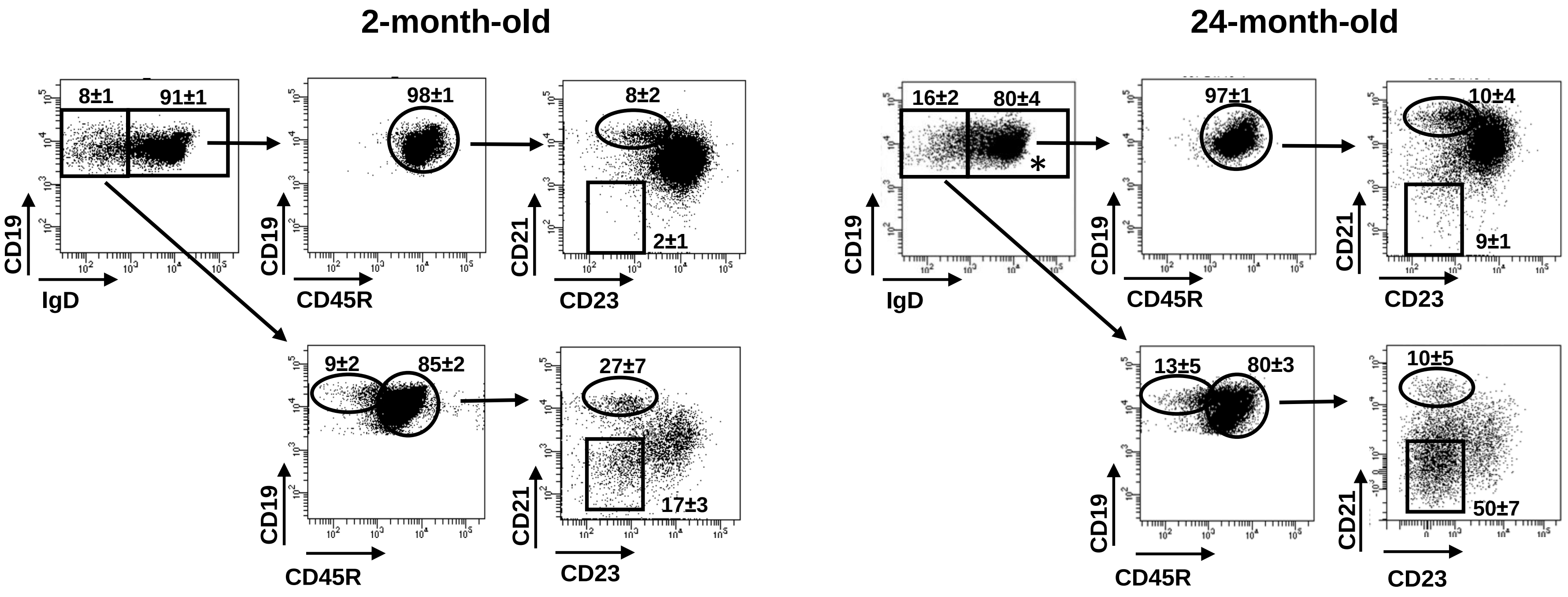

Figure S2: Caracterization of different B-lymphocyte populations on 2-month-old and 24-month-old C57BL/6. Different B-cell subsets were analysed on splenic preparations by electronically gating CD19^{+} cells. Stainings were performed as described on Figure 1. * P > 0.05
