# Supplementary material for: Altered marginal zone and innate-like B cells in aged senescence-accelerated SAMP8 mice with defective IgG1 responses
Source: Cell Death Dis. 2017 Aug 17;8(8):e3000–. doi: 10.1038/cddis.2017.351 (PMC5596542; doi:10.1038/cddis.2017.351)
Supplement: Supplementary Figure S3 [file cddis2017351x3.pdf]

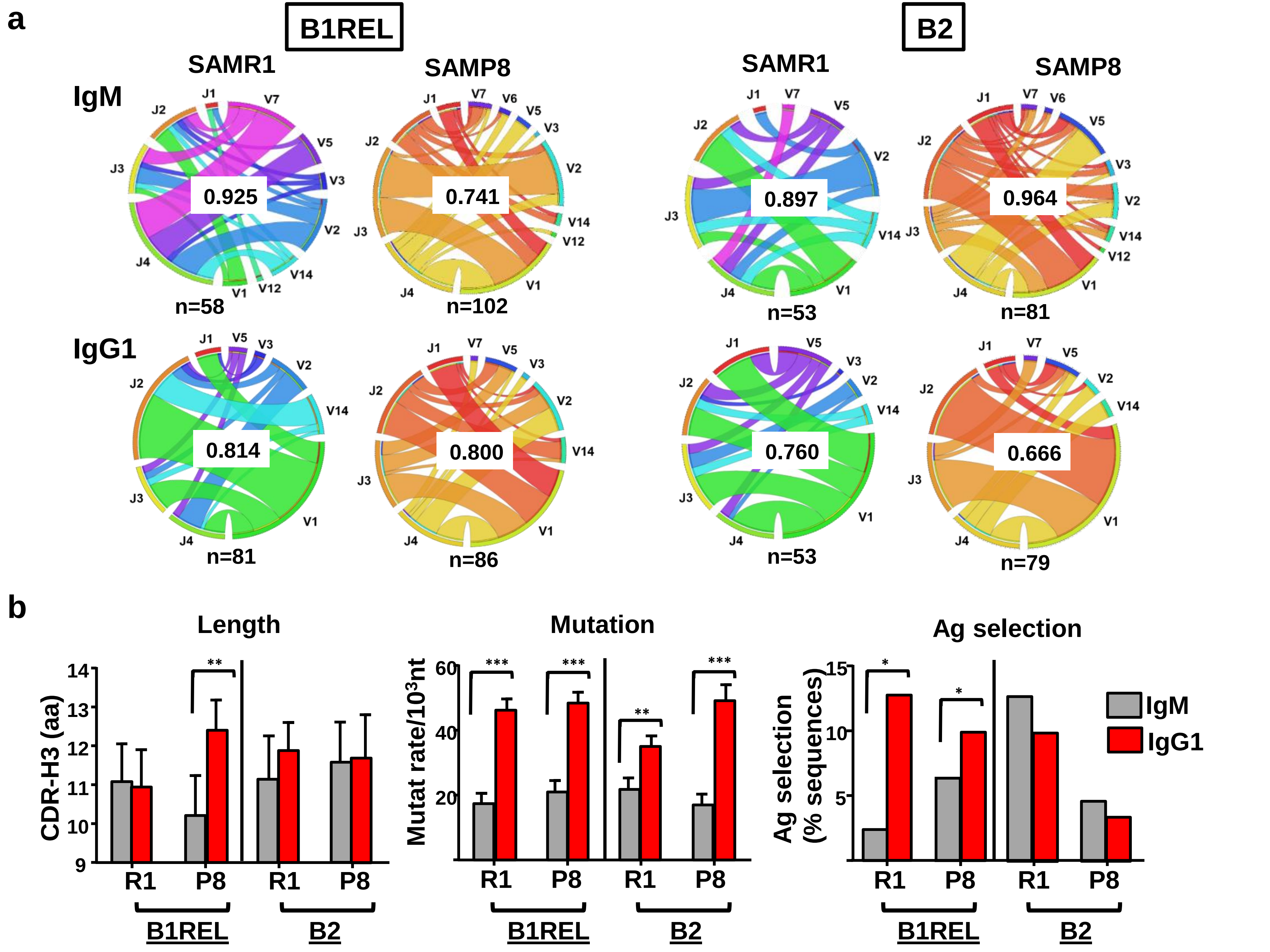
a
B1REL
B2
SAMR1
SAMP8
IgM
IgG1
0.925
0.741
0.897
0.964
0.814
0.800
0.760
0.666
n=58
n=102
n=53
n=81
n=81
n=86
n=53
n=79
b
Length
Mutation
Ag selection
CDR-H3 (aa)
Mutat rate/10³nt
Ag selection (% sequences)
IgM
IgG1
R1
P8
B1REL
B2

Supplementary Figure S3: Analysis of the V_HJ_H repertoire displayed in IgM and IgG1 sequences from 10-month-old SAMP8 and SAMR1 mice. Analyses were performed using the strategy and software described in Materials and Methods.[38](**a**) Circle graph representations of the $V_HJ_H$usage from IgMand IgG1 sequences found in B1REL and B2 cells. The strips inside the circles indicate the V_H to J_H rearrangements, and their thickness is proportional to the frequency at which each rearrangement was found.The numbers inside the circles represent the clonal diversity in each group of sequences, as the ratio between the number of clonotypes relative to the unique sequences in the group. Clonality of the sequences analyzed was studied on sequences using the same V and J genes, identical CDR-H3 length and less than 10% differences on CDR-H3 in nucleotide sequence. Ambiguous and uncompleted sequences were discarded.Numbers below each circle are the number of sequences analyzed. (**b**) The bar graph in the left represents the length (as amino acids) of the CDR-H3 region found for each sequence; the graph in the middle shows the mutation rate determined for each group of sequences; the right graph displays the Ag selection rate as determined by using the algorithm for the binomial distribution method of Chang and Casali.[53] Data are the mean ± SEM,and comparisons were performed by unpaired two-tailed Student´s *t*-test, except for the Ag selection data. In this case, data are the percentages of sequences found above the upper confidence limits of the distributions, and the comparisons were made by using the Chi-squared test. *$P< 0.05$; **$P< 0.01$; ***$P< 0.001$. n=593 sequences, from 7 aged SAMP8 and 6 SAMR1 mice.
