# Supplementary material for: Altered marginal zone and innate-like B cells in aged senescence-accelerated SAMP8 mice with defective IgG1 responses
Source: Cell Death Dis. 2017 Aug 17;8(8):e3000–. doi: 10.1038/cddis.2017.351 (PMC5596542; doi:10.1038/cddis.2017.351)
Supplement: Supplementary Figure S4 [file cddis2017351x4.pdf]

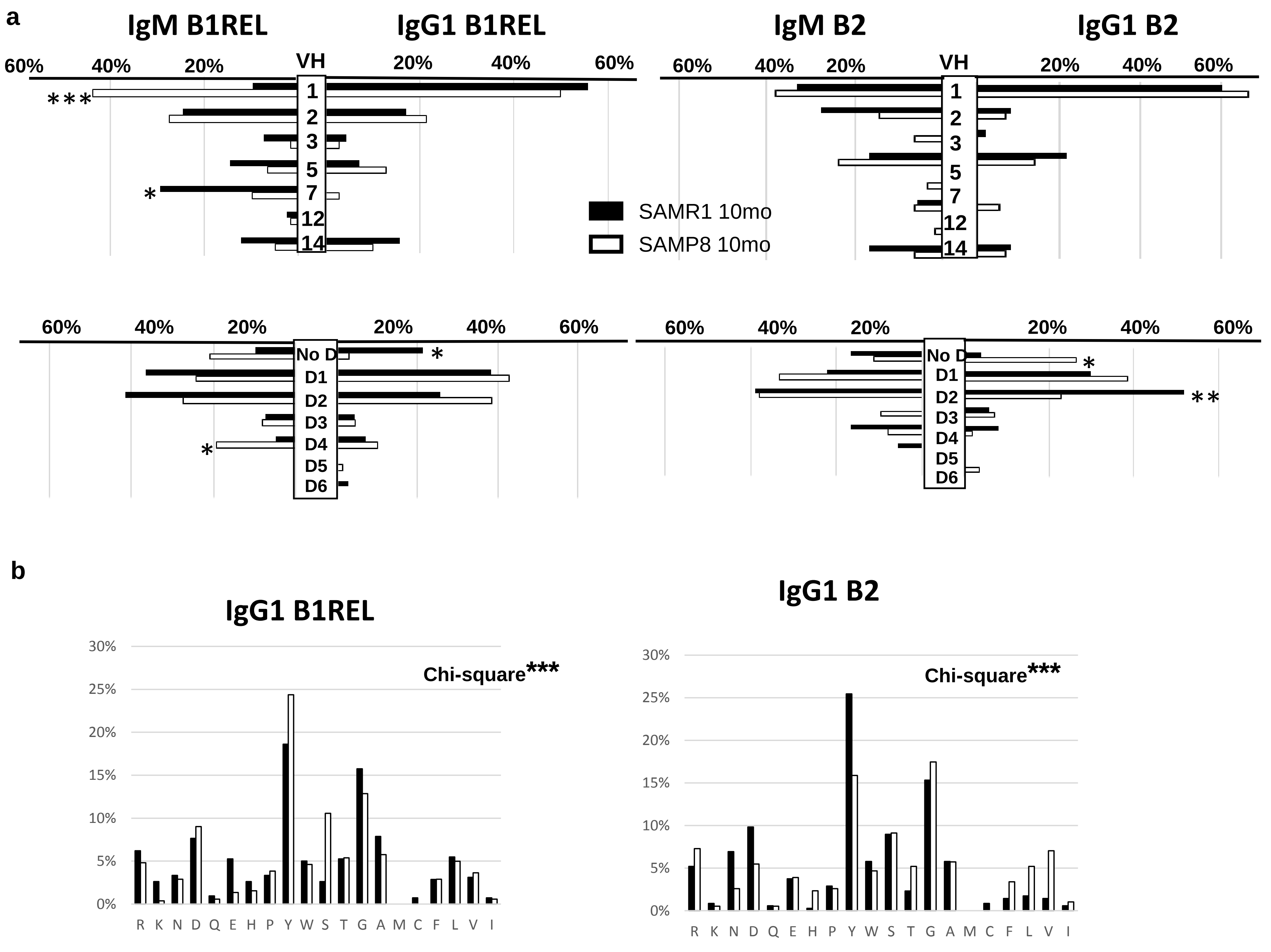

a
IgM B1REL
IgG1 B1REL
IgM B2
IgG1 B2
VH
60%
40%
20%
1
2
3
5
7
12
14
***
*
SAMR1 10mo
SAMP8 10mo
No D
D1
D2
D3
D4
D5
D6
**
b
IgG1 B1REL
IgG1 B2
Chi-square***
30%
25%
20%
15%
10%
5%
0%
R K N D Q E H P Y W S T G A M C F L V I

c

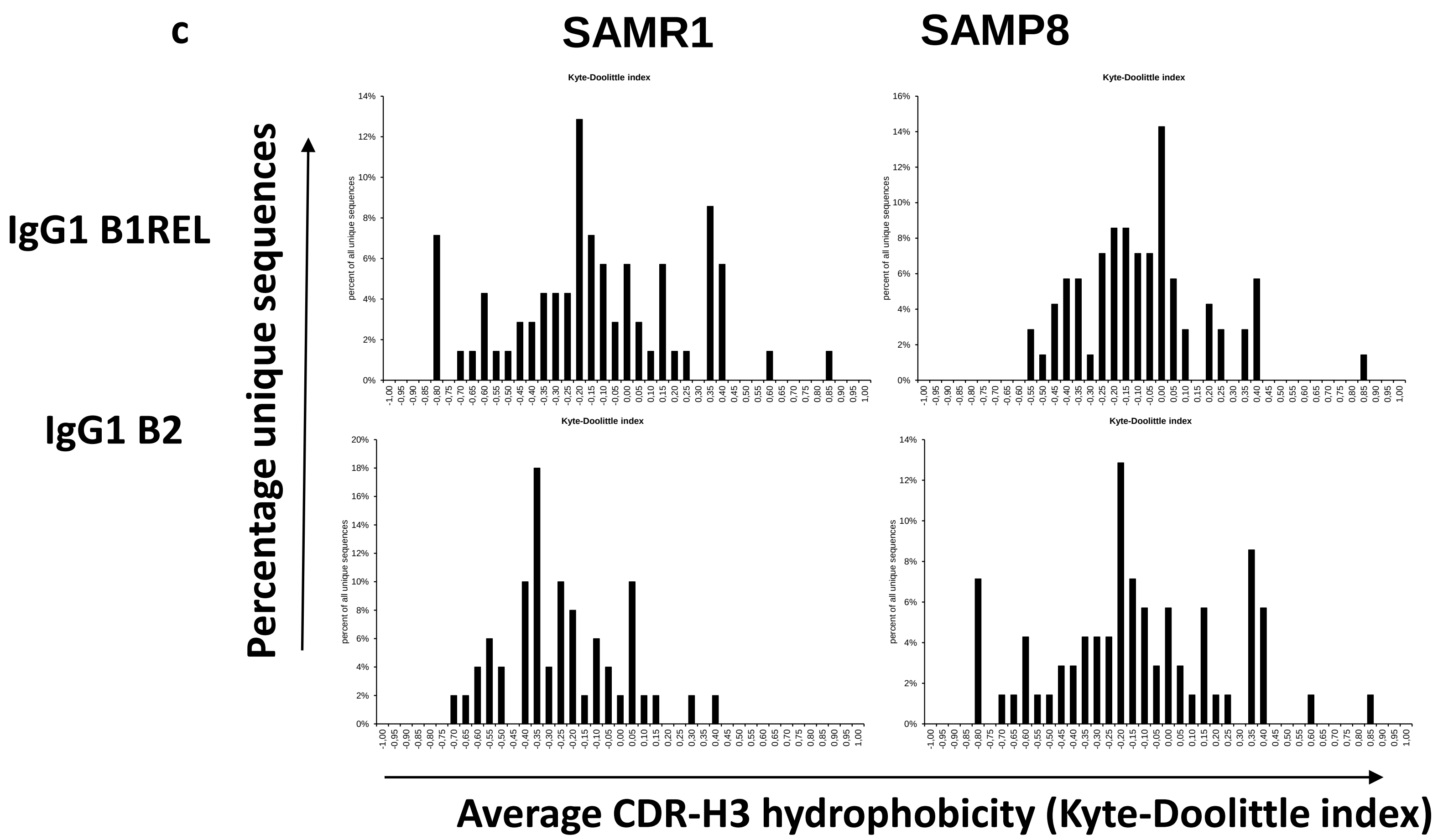

Kyte-Doolittle index			Student´s *t*-test
	SAMR1	SAMP8	R1/P8
B1REL	-0.16	-0.10	***
B2	-0.26	-0.04	***

Figure S4: V_H, D_H rearrangements and AA usage of the sequences obtained from B1REL and B2 from aged SAMR1 10mo and SAMP8 10mo mice. (**a**) V_H and D_H usage expressed as percentages of the total samples analysed. Statistical analyses were performed using the Chi-square test for contingency tables as indicated below the histograms: *$p < 0.05$, **$p < 0.01$. (**b**) Overall amino acid frequencies distribution in the CDR-H3 loop of the indicated samples. Amino acids are arranged by polarity from arginine (left) to isoleucine (right). Comparisons on the AA usage distribution between SAMR1 and SAMP8 in CD3-H3 loop of IgG1 sequences from the B1REL and the B2 cell populations were tested for statistical significance using the Chi-square test, significant differences between both groups were found (****$p < 0.001$. (**c**) Average hydrophobicity of the CDR-H3 loop from the groups shown in the figure was calculated using the normalized Kyte-Doolittle index. Data were obtained from the IgAT software (*Eisenberg et al*.). Comparisons between groups were studied using the two-tailed Student´s *t*-test.

Eisenberg, D. 1984. Three-dimensional structure of membrane and surface proteins. *Annu Rev Biochem* 53:595-623.
