# Supplementary material for: Altered marginal zone and innate-like B cells in aged senescence-accelerated SAMP8 mice with defective IgG1 responses
Source: Cell Death Dis. 2017 Aug 17;8(8):e3000–. doi: 10.1038/cddis.2017.351 (PMC5596542; doi:10.1038/cddis.2017.351)
Supplement: Supplementary Figure S5 [file cddis2017351x5.pdf]

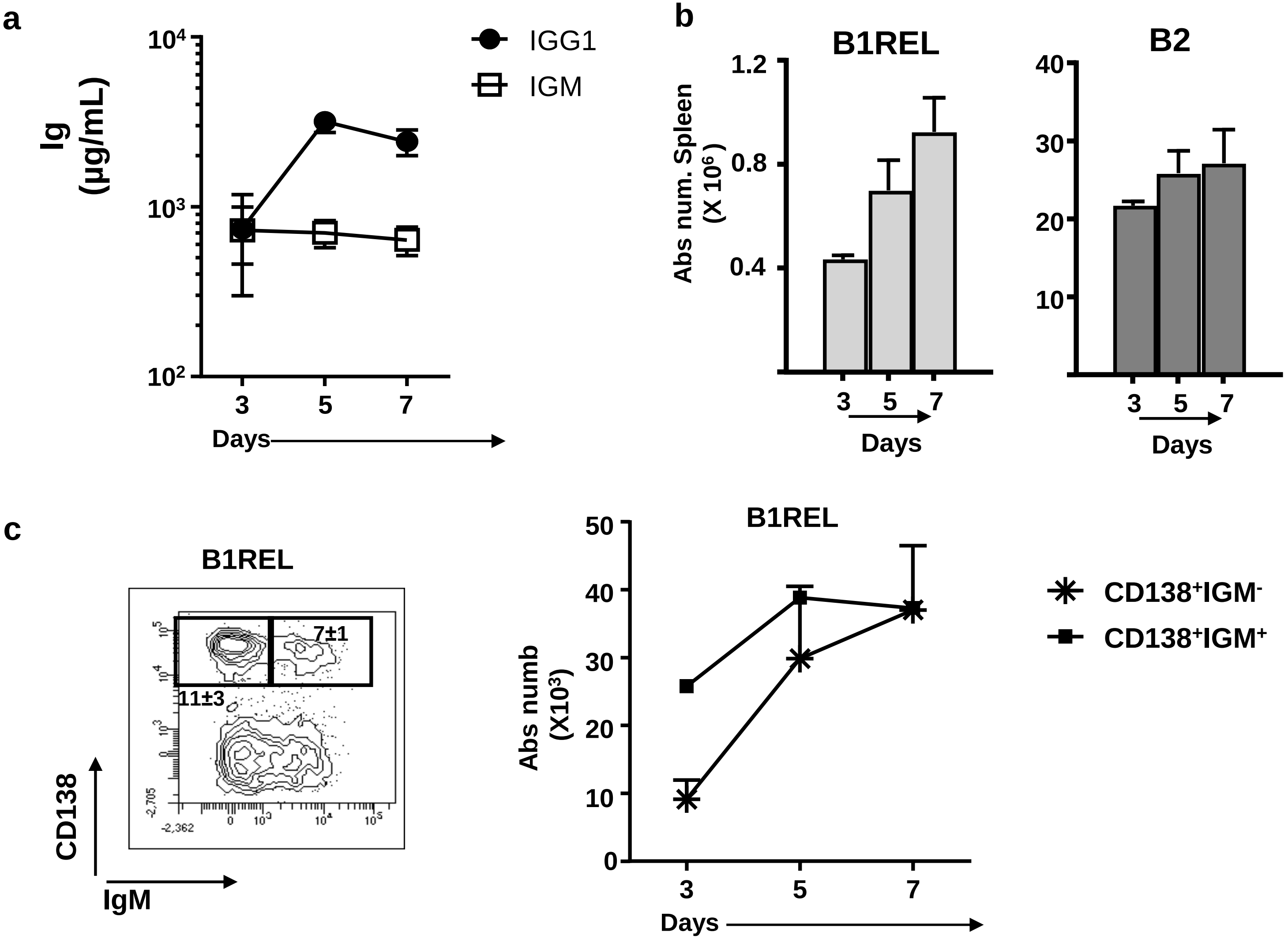

Figure S5: In vivo stimulation of BALB/c 2-month-old with LPS. Mice were injected i.p,. with LPS as described in Materials and Methods. Sera and splenocytes were collected on days 3, 5 and 7 after immunization. (**a**) ELISA sera determination for IgM and IgG1. (**b**) Quantitation of B1REL cells (left graph) and B2 cells (right graph) as described in Fig. 1b. (**c**) B1RELquantitation of $CD138^{+}IgM^{+}$ and $CD138^{+}IgM^{-}$ cells (n = 3).
