# Supplementary material for: Altered marginal zone and innate-like B cells in aged senescence-accelerated SAMP8 mice with defective IgG1 responses
Source: Cell Death Dis. 2017 Aug 17;8(8):e3000–. doi: 10.1038/cddis.2017.351 (PMC5596542; doi:10.1038/cddis.2017.351)
Supplement: Supplementary Table S1 [file cddis2017351x6.pdf]

Table S1. Antibodies used on flow cytometric studies

MoAbs	Clone	Label	Company
CD4	GK1.5	APC	BioLegend
CD5	53-7.3	APC	BioLegend
CD9	MZ·	Alexa-Fluor647	BioLegend
CD11b	M1/70	PECy7,APCCy7	BioLegend
CD19	1D3	PE, PECy7, Violet-421	e-biosciences, BioLegend
CD21	7E9	FITC, APC	BioLegend
CD23	B3B4	PECy7	BioLegend
CD38	90	APC	BioLegend
CD45R/B220	RA3-6B2	FITC, PE	BioLegend
CD138	MI15	APC, PE, BIO	BD
CD169	MOMA-1	BIO	Abcam
CXCR5 (CD185)	L138D7	Violet-421	BioLegend
GL-7	GL-7	PE	BioLegend
Gr-1	RB6-8C5	PECy7	BioLegend
PD1 (CD279)	29F.1A12	BIO	BioLegend
TACI (CD267)	8F10	PE	BioLegend
BAFFR(CD268)	7H22-E16	PE	BioLegend
Isotype controls		FITC/PE/APC/ PECY7/APCCy7/Violet	BD
Streptavidin		FITC/PE/APC/APCCy7	BioLegend
IgG1	A85-1	BIO	BD
IgM	331.13, RMM1	FITC, APCCY7/PECy7	In house, BioLegend
IgD	11-26c.2a	Alexa-Fluor488	BioLegend

BIO, biotin; FITC, fluorescein isothiocyanate; PE, phycoerythrin; APC, allophycocyanin,
